# Supplementary material for: Empirical methods for controlling false positives and estimating confidence in ChIP-Seq peaks
Source: BMC Bioinformatics. 2008 Dec 5;9:523. doi: 10.1186/1471-2105-9-523 (PMC2628906; doi:10.1186/1471-2105-9-523)
Supplement: Additional File 1 — A variety of html documents from the USeq web site detailing the available applications, their best usage, output file type descriptions, command line menus, etc. [file 1471-2105-9-523-S1.zip › USeqUserGuides/JavaDoc.zip!/JavaDoc/trans/misc/GrGraph.html]

GrGraph


|  |  |  |
| --- | --- | --- |
| **Overview** \| **Package** \| **Class** \| **Use** \| **Tree** \| **Deprecated** \| **Index** \| **Help** | |  |
| **PREV CLASS**   **NEXT CLASS** | **FRAMES**    **NO FRAMES**     **All Classes** |  |
| SUMMARY: NESTED \| FIELD \| CONSTR \| METHOD | DETAIL: FIELD \| CONSTR \| METHOD |  |


---


## trans.misc Class GrGraph

```
java.lang.Object
  trans.misc.GrGraph
```

---

``` public class GrGraph extends java.lang.Object ```

Container for holding info related to a xxx.gr file.

---

**Constructor Summary**

`GrGraph(java.lang.String chromosome,
java.lang.String genomeVersion,
Gr[] grs)`

`GrGraph(java.lang.String chromosome,
java.lang.String genomeVersion,
int[] basePositions,
float[] values)`


| **Method Summary** | |
| --- | --- |
| `int[]` | `getBasePositions()` |
| `java.lang.String` | `getChromosome()` |
| `java.lang.String` | `getGenomeVersion()` |
| `float[]` | `getValues()` |
| `void` | `loadGrArray(Gr[] grs)` |
| `static GrGraph[]` | `loadSgrFile(java.io.File sgrFile)`             Loads an sgr file returning chromosome specific GrGraphs. |
| `void` | `setBasePositions(int[] basePositions)` |
| `void` | `setChromosome(java.lang.String chromosome)` |
| `void` | `setGenomeVersion(java.lang.String genomeVersion)` |
| `void` | `setValues(float[] values)` |
| `boolean` | `writeGrFile(java.io.File name)`             Writes to the file base tab value. |

**Methods inherited from class java.lang.Object**

`equals, getClass, hashCode, notify, notifyAll, toString, wait, wait, wait`

**Constructor Detail**

### GrGraph

```
public GrGraph(java.lang.String chromosome,
               java.lang.String genomeVersion,
               int[] basePositions,
               float[] values)
```

---


### GrGraph

```
public GrGraph(java.lang.String chromosome,
               java.lang.String genomeVersion,
               Gr[] grs)
```


**Method Detail**

### loadGrArray

```
public void loadGrArray(Gr[] grs)
```

---


### loadSgrFile

```
public static GrGraph[] loadSgrFile(java.io.File sgrFile)
```

:   Loads an sgr file returning chromosome specific GrGraphs.

---


### writeGrFile

```
public boolean writeGrFile(java.io.File name)
```

:   Writes to the file base tab value.
    It's strongly recommended to use the chromosome and genomeVersion in the file name!

---


### getBasePositions

```
public int[] getBasePositions()
```

---


### setBasePositions

```
public void setBasePositions(int[] basePositions)
```

---


### getChromosome

```
public java.lang.String getChromosome()
```

---


### setChromosome

```
public void setChromosome(java.lang.String chromosome)
```

---


### getGenomeVersion

```
public java.lang.String getGenomeVersion()
```

---


### setGenomeVersion

```
public void setGenomeVersion(java.lang.String genomeVersion)
```

---


### getValues

```
public float[] getValues()
```

---


### setValues

```
public void setValues(float[] values)
```


---


|  |  |  |
| --- | --- | --- |
| **Overview** \| **Package** \| **Class** \| **Use** \| **Tree** \| **Deprecated** \| **Index** \| **Help** | |  |
| **PREV CLASS**   **NEXT CLASS** | **FRAMES**    **NO FRAMES**     **All Classes** |  |
| SUMMARY: NESTED \| FIELD \| CONSTR \| METHOD | DETAIL: FIELD \| CONSTR \| METHOD |  |


---
